# Non-clinical managers: the key to a successful eye programme

**Published:** 2014

**Authors:** Edson Mwaipopo

**Affiliations:** Deputy director: Kilimanjaro Centre for Community Ophthalmology Tanzania LTD. E-mail: eeliah@kcco.net

**Figure:**
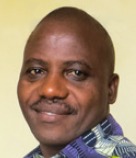
Edson Mwaipopo

The importance of management in an eye care programme should not be underestimated. It is hard to imagine how a busy ophthalmologist could attend to her or his patients, as well as doing all of the tasks needed to manage a successful eye department, eye hospital or hospital-based VISION 2020 eye programme. Some of these tasks are set out below.

- Planning and organising clinic-based and outreach activities.
- Receiving and issuing supplies, including for operating theatre, the outpatient department, and refractive error services.
- Making regular inventory reports and reordering when necessary to ensure that supplies are in stock.
- Keeping track of accounting for the hospital or overall eye care programme and producing reports, including a cost recovery report.
- Preparing and submitting reports to donors and partner organisations.
- Ensuring that the programme vehicle is maintained properly.
- Identifying and liaising with potential donors/ sponsors who can support the hospital and/or VISION 2020 activities in the region
- Managing human resource issues, including recruitment, annual leave/ vacations and deployment.

It makes sense to employ a programme manager to carry out the above tasks. If there is a good manager in the team, the ophthalmologist can meet regularly with her or him to review reports and agree budgets. The ophthalmologist can dedicate the rest of her or his time to examining and treating patients.

## Finding a good manager

There is no specific academic background needed, but it is preferable to recruit someone with some management and administrative training. It is not recommended to train and recruit clinical staff to this post. This is a full time job and there is a very limited number of trained clinical staff in low- and middle-income countries; they should not be taken away from their clinical duties. It is easier and more economical to train a non-clinical staff member as a programme manager.

It is ideal if programme managers are recruited and employed by the central hospital, rather than independently (by the eye department alone). This will help managers to be accountable and will make it easier for them to work with other hospital staff members, e.g. accountants, store keepers, the hospital administrator and the matron, all of whom play an important role in the success of the eye programme or eye department.

## Training and capacity building

After recruitment, it is important that the new manager learns as much as possible about eye care, which can be done at the eye department. The new manager needs to understand how the departmental systems work internally as well as within the wider health system.

It may be helpful to assign one clinical staff member to assist the new manager in the learning process. There are easy-to-understand reading materials available on eye health, leading causes of blindness and their management. The *Community Eye Health Journal* is recommended!

The manager must also learn about international and World Health Organization (WHO)-led actions such as VISION 2020 and the Global Action Plan for Universal Eye Health 2014–19. Eye care service delivery planning, leadership skills, team building, partnership building, the basics of financial and human resources management, and bridging strategies to connect hospitals and communities are all essential for the task of management. Some training is available: courses covering these subjects are run by KCCO International, LAICO/Aravind, the University of Cape Town, the London School of Hygiene and Tropical Medicine, and others (see page 40).

It should be noted that the learning and capacity building process must be ongoing. The manager needs to learn every day, even after being fully employed. It will take sometime before the new manager will understand all the aspects of her or his job.

## Mentoring

This involves someone with experience in eye care management keeping in contact with a new manager to assist and advise her or him. Mentoring is a training and capacity building process, and new managers are lucky if they can take advantage of this. Mentoring is ongoing, with more frequent contacts and communications at the beginning and fewer being needed as the manager gains the confidence and skills to work independently.

A manager can make a big difference in an eye care programme, especially one that reaches proactively into the community to provide services to those who would not come to hospital on their own. The enjoyment of the clinical staff when they are free to do what they do best, with lots of patients, in an efficiently run system, is also a large bonus for an eye programme!

**Figure:**
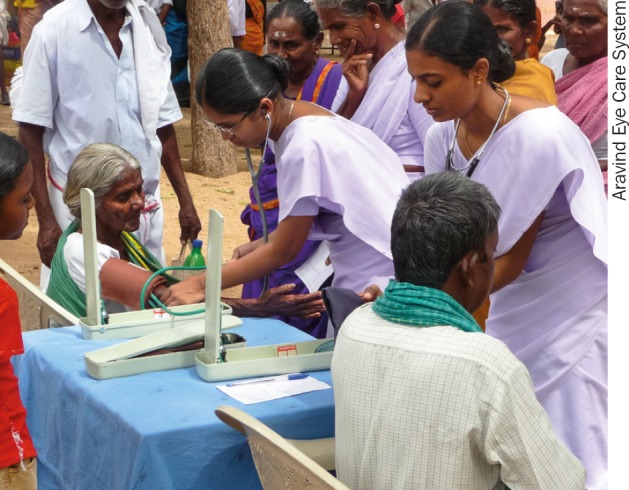
When an eye programme has a non-clinical manager, organising outreach activities will become one of his or her main responsibilities.

## View from an ophthalmologist

**Figure:**
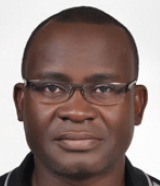


**Asiwome Seneadza** is head of Kitwe Central Hospital's eye department in Zambia. The hospital is run by the government and is a tertiary referral centre for the northern part of Zambia, with its population of 4.7 million. Currently, it is the only centre in Zambia offering paediatric and retinal services.

### Why did you employ a manager?

I realised early on that my performance would be affected if I had to take on both the clinical and administrative responsibilities involved in managing the eye department. Although I still have administrative responsibilities, my key focus is the clinical excellence of our department.

### What were you hoping the manager would do for the eye department?

First and foremost, I wanted the manager to have a sense of ownership! Fortunately, he acquired this early, partly because we set targets together – at that time it was targets for cataract surgery. He looked into how we could improve our finances, conduct outreach, place orders, and write reports.

### How did you find a good manager? What did you look for?

Initially, the key focus for me was improving our financial accounting. As we didn't have funding at the time, I selected a very good accountant from the central hospital to help us part time. Fortunately he picked up most of the eye work very fast! He also attended a 6-week management course at Aravind in India, and has recently attended a refresher course. I think the ideal manager is very dedicated, hardworking and, above all, honest. Having very good interpersonal skills is also important.

### How do you divide your responsibilities?

He does the narrative and financial reporting, supervises support staff, and communicates with our partners. I look after the clinical side of things and come up with new ideas which he will implement after discussing them with the rest of the team.

### How do you ensure a good working relationship?

Frequent and good communication, at least three times per day. There is a strong sense of teamwork and shared responsibility. When one of us is unable do a task, the other takes it up. We regularly review our activities and know who is responsible for what.

### What were the biggest challenges for you both?

There is no government recognition for managers in eye care, so there is no career path. Finding money is therefore challenging and at the moment our manager's salary is partly dependent on NGO funding.

### What are the benefits of having a manager? Would you recommend it to others?

First of all, it is impossible to do all the necessary administrative work and remain a clinician. Clinicians can be leaders but not managers. Managers are crucial for any programme to run successfully!

## View from a manager

**Figure:**
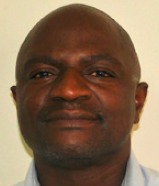


Organising outreach activities is one of the most important responsibilities of **Winston Mbao**, the non-clinical manager at Kitwe Central Hospital. Here he explains how its outreach programme has helped to increase the cataract surgical output at the hospital.

Before I was appointed in 2003, the hospital performed around 500 cataract operations per year. The current output is 2,500 per year, mainly due to outreach.

Zambia is sparsely populated and people often cannot afford transport to an eye health facility. We visit a different community every week to screen people and then bring those who need surgery to our hospital. We also provide return transport.

Eight times a year one of our surgical teams will also go and ‘camp’ at another district hospital. They screen people at different local health centres, transport those who need surgery to the hospital, and take them back afterwards. This involves a lot of planning! As a manager, one of my most important responsibilities is to organise these outreach activities, so services reach where the need is greatest.

*For more information, please contact Winston Mbao at Kitwe Central Hospital. Email:* cespkch@yahoo.com

**Case study: Madagascar**

The Antsinanana VISION 2020 programme was established in June 2010 and is a joint effort between the Madagascan Ministry of Health, Kilimanjaro Centre for Community Ophthalmology (KCCO), Swiss Lions and Lions Sight First Madagascar.

The region has two facilities providing secondary eye care services: the Atsinanana Regional Hospital in Toamasina and the Vatomandry District Hospital.

**Figure:**
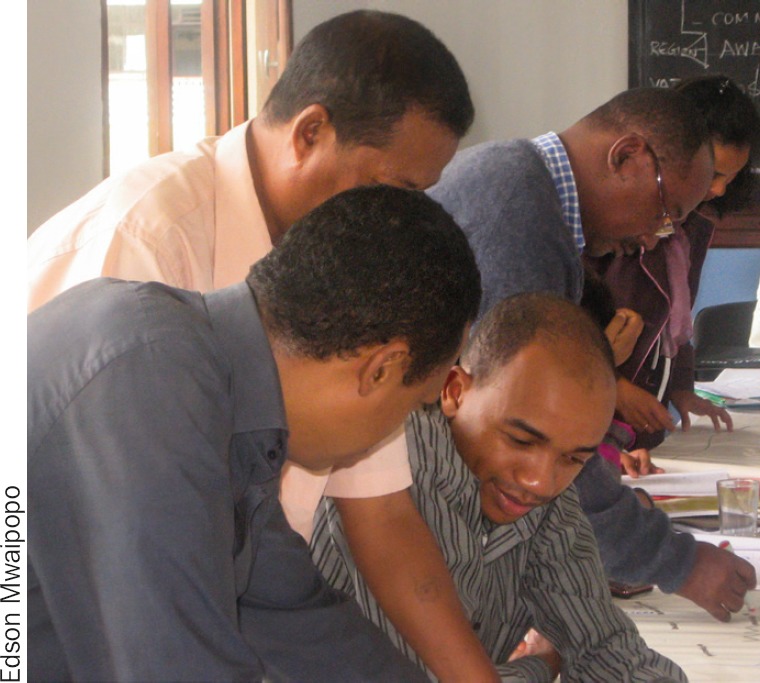
A planning meeting

Initially, there were no managers – either for the regional eye care programme or the two hospitals. The ophthalmic staff had to perform all clinical and non-clinical duties. Although both hospitals had basic equipment to enable them to perform surgery, the two centres performed only 481 cataract operations per year (in 2010). Services were limited to those patients who could pay for their own transport to the two facilities.

The subsequent employment of a full-time non-clinical manager gave the clinical staff more time to do clinical work. Staff members enjoyed this change and were able double the number of cataract operations to 1,068 in 2011, while in 2013 they performed a total of 1,728 cataract operations.

Due to strong advocacy by the manager there is now also more Ministry of Health involvement. Reports are prepared and submitted on time and the role of each staff member has been carefully spelled out.

